# Collaborating on the 100th Day of School: Gerontology, Sociology, Education, and Social Work, Oh My!

**DOI:** 10.1093/geroni/igaf122.877

**Published:** 2025-12-31

**Authors:** Cynthia Hancock, Tina Newsham, Daniel Alston, Lisa Borrero, Katherina Nikzad-Terhune, Sarah Tesar, Elizabeth Fugate-Whitlock

**Affiliations:** The University of North Carolina at Charlotte, Charlotte, North Carolina, United States; The University of North Carolina at Wilmington, Wilmington, North Carolina, United States; The University of North Carolina at Charlotte, Charlotte, North Carolina, United States; University of Indianapolis, Indianapolis, Indiana, United States; Northern Kentucky University, Highland Heights, Kentucky, United States; The University of North Carolina at Charlotte, Charlotte, North Carolina, United States; The University of North Carolina at Wilmington, Wilmington, North Carolina, United States

## Abstract

Given that some celebrations of the 100th day of school involve having young children dressing up “like a 100-year-old,” a gerontologist and a social worker with expertise in gerontology questioned the ageist ideas this activity could reinforce. Recognizing that children adopt beliefs about various social groups at an early age, we saw the 100th day of school as an opportunity to teach accurate and positive information about aging and older adulthood rather than perpetuate ingrained stereotypes. The small team recognized that additional expertise was needed and reached out to others, including a sociologist, other social workers with gerontology expertise, an expert in health and aging, and an elementary educator with expertise in diversity and inclusion. The interdisciplinary team has collaborated for over three years to provide pre-K-2 teachers with evidence-based educational materials related to aging (centenarians in particular). We created a toolkit about aging and ageism to give teachers options for celebrating the 100th day of school in a way that reinforces academic content while supporting age-inclusivity. In this presentation, we share how the team was formed, describe strategies we use for collaborating, and identify the benefits of working on an innovative interdisciplinary collaboration to help eliminate ageism.

